# Vaccine Confidence and Uptake of the Omicron Bivalent Booster in Tennessee: Implications for Vulnerable Populations

**DOI:** 10.3390/vaccines11050906

**Published:** 2023-04-27

**Authors:** Donald J. Alcendor, Patricia Matthews-Juarez, Duane Smoot, Alexis Edwards, James E. K. Hildreth, Paul D. Juarez

**Affiliations:** 1 Department of Microbiology, Immunology, and Physiology, Center for AIDS Health Disparities Research, School of Medicine, Meharry Medical College, 1005 Dr. D.B. Todd Jr. Blvd., Nashville, TN 37208, USA; 2 Department of Family & Community Medicine, Meharry Medical College, 1005 D.B. Todd Jr. Blvd., Nashville, TN 37208, USA; 3 Department of Internal Medicine, School of Medicine, Meharry Medical College, 1005 D.B. Todd Jr. Blvd., Nashville, TN 37208, USA; 4 Office of Minority Health, Division of Health Disparities, Tennessee Department of Health, Nashville, TN 37208, USA

**Keywords:** COVID-19, boosters, Tennessee, bivalent omicron booster, vaccine hesitancy

## Abstract

The COVID-19 Omicron variant and its subvariants are now the dominant variants circulating in the US. Therefore, the original COVID-19 vaccine cannot offer full protection. Instead, vaccines that target the spike proteins of the Omicron variants are warranted. Hence, the FDA recommended the development of a bivalent booster. Unfortunately, despite the safety and immunogenicity of the Omicron bivalent boosters from Pfizer and Moderna, uptake in the US has been poor. At this time, only 15.8% of individuals in the US aged five and older have received the Omicron bivalent booster (OBB). The rate is 18% for those aged 18 and older. Poor vaccine confidence and booster uptake are often fueled by misinformation and vaccine fatigue. These result in more problems associated with vaccine hesitancy, which are particular prevalent in Southern states in the US. In Tennessee, the OBB vaccination rate for eligible recipients is only 5.88% at time of writing (16 February 2023). In this review, we discuss (1) the rationale for developing the OBBs; (2) the efficacy and safety of the bivalent boosters; (3) the adverse events associated with these boosters; (4) vaccine hesitancy associated with the OBBs uptake in Tennessee; (5) implications for vulnerable populations, disparities in uptake of OBBs in Tennessee, and strategies to improve vaccine confidence and OBB uptake. In support of public health, it is essential that we continue to provide education, awareness, and vaccine access to the vulnerable and medically underserved populations in Tennessee. Receiving the OBBs is the most effective method to date of protecting the public against severe COVID disease, hospitalization, and death.

## 1. Introduction

Currently, four COVID-19 vaccines are in use in the US, either under a Biologics License Application or authorized under an emergency use authorization (EUA) by the FDA [1]. The Advisory Committee on Immunization Practices (ACIP) has approved the use of: (1) the two- or three-dose monovalent mRNA BNT162b2 (Pfizer-BioNTech, Comirnaty, New York, NY, USA) COVID-19 vaccine; (2) the two- or three-dose monovalent mRNA mRNA-1273 (Moderna, Spikevax, Cambridge, UK) COVID-19 vaccine; (3) the single-dose adenovirus vector-based Ad26.COV.S (Janssen [Johnson & Johnson], Beerse, Belgium) COVID-19 vaccine; and (4) the two-dose adjuvant-, protein subunit-based NVX-CoV2373 (Novavax, Gaithersburg, MD, USA) COVID-19 vaccine [2]. The number of doses recommended is based on the age and condition of the immune system of the recipient. For additional protection, the FDA has amended the EUAs to allow eligible individuals to obtain COVID-19 booster doses (1). Because COVID-19 vaccines have demonstrated decreased effectiveness against the Omicron variant (B.1.1.529) of SARS-CoV-2, bivalent boosters with equal components of the ancestral and Omicron strains are considered for the express purpose of improving protection (2) [3]. The Moderna mRNA bivalent booster is preferred for individuals who are moderately to severely immunocompromised because it showed a more robust immune response in this population than the Pfizer bivalent mRNA vaccine [3]. The Pfizer bivalent booster is approved for use in individuals aged 5 to 17 years. At the time of writing, we identified only 48 publications in PubMed that focus on the bivalent Omicron boosters for COVID-19. Interestingly, we did not identify any publication on vaccine confidence and uptake of the Omicron bivalent booster (OBB) in Tennessee. The state of Tennessee ranks 9th per 100,000 persons for uninsured persons compared to other states in the US [4]. The Kaiser Family Foundation indicates that more than 11% of the Tennessee population of approximately 800,000 residents are uninsured [5]. Tennessee had the 4th highest age-adjusted death rate per 100,000 due to COVID-19 among the 17 Southern states during the 3rd quarter of 2022, the most recent data reported by the CDC [6]. As of 10 March 2023, Tennessee ranks 8th in the US in COVID-19 death rates per 100,000 persons [7]. Southern states in the US consistently rank among the worst for health and wellness in the US. Southern states have the highest rates per 100,000 of premature deaths due to chronic conditions that can go untreated due to poverty, health disparities, and institutionalized racism compared to other states in the US [8,9,10]. There are higher levels of obesity in Southern states and individuals are more likely to smoke and live a sedentary lifestyle [11]. There is less access to healthcare providers in the South compared to other regions in the US [12]. The high incidence of chronic diseases found in the South are associated with comorbidities that predispose individuals to the most severe complications of COVID-19 and the South is the region where COVID-19 vaccine uptake is the lowest in the US [13]. At the time of writing, only 15% of individuals eligible for the updated bivalent Booster in the US have received it [14]. In Tennessee, only 5.88% of eligible individuals have received the updated bivalent booster [15].

Here, we examine (1) the rationale for developing the OBB; (2) the efficacy and safety of the bivalent boosters; (3) the adverse events associated with these boosters; (4) vaccine hesitancy associated with the boosters; (5) implications for vulnerable populations; and (6) strategies to improve vaccine confidence and uptake of the boosters with a focus on the state of Tennessee.

## 2. The Rationale for Development of the Omicron Bivalent Boosters

**Figure 1:**
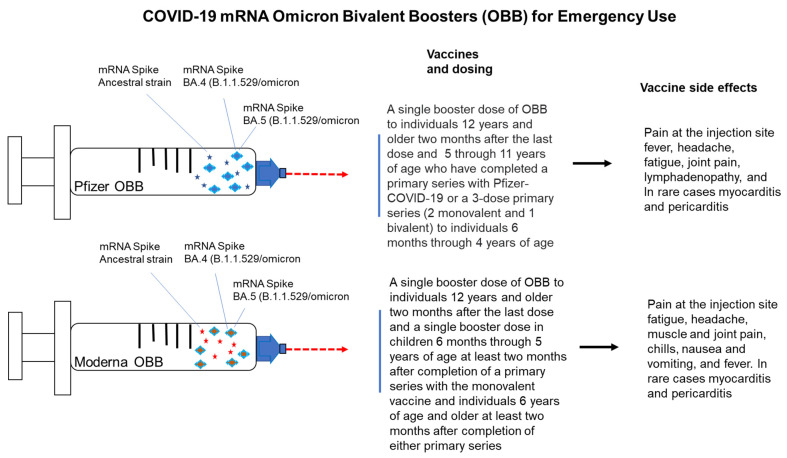
Development of the omicron bivalent boosters.

The emergence of more contagious variants of COVID-19 that can escape detection by our immune system warranted the development of a booster that can confer protection against these variants [16]. The Omicron variant and its subvariants can infect even individuals who have been vaccinated and boosted with monovalent vaccines. Due to the emergence of the omicron BA.4 and BA.5 variants, the FDA recommended that vaccines against the BA.4/BA.5 variants be developed [17,18]. In June 2022, the FDA requested that both Pfizer and Moderna develop a bivalent vaccine consisting of equal amounts of mRNAs that encode the spike protein from the ancestral SARS-CoV-2 strain as well as the spike protein from the Omicron BA.4/BA.5 subvariants [19] (Figure 1). It is important to note that BA.4 and BA.5 subvariants do not differ in their spike proteins or their receptor-binding domains (RBDs), but instead differ in other viral proteins [20]. The updated COVID-19 boosters would retain the ability to neutralize the ancestral viral strain and provide a more robust immune response against the circulating Omicron subvariants. These updated boosters would have the potential to neutralize future variants and extend the duration of immune protection [19]. The FDA authorized the Pfizer and Moderna OBBs on 31 August 2022 [21]. These boosters contain equal amounts of mRNAs encoding the ancestral strain spike protein and the spike proteins from the BA.4 and BA.5 strains of Omicron variant (Figure 1). These boosters were authorized for emergency use as a single dose to be given at least two months after a primary or booster vaccination [21]. On 1 September 2022, the bivalent boosters from Pfizer and Moderna replaced the monovalent boosters for individuals aged 12 years and above in the US and in other countries [22]. Guidance for dosing and potential side effects of these boosters were also presented in Figure 1.

## 3. Efficacy and Safety of the Bivalent Omicron Boosters

In an open-label phase 2/3 trial Chalkias et al., show that the Moderna bivalent booster produces a robust immune response resulting in antibody titers that are likely to be protective against multiple COVID-19 variants [23]. An immunogenicity objective was to demonstrate superior antibody responses compared to the previously authorized mRNA-1273 50-µg booster. Immune responses 28 days and 180 days after the 50-µg mRNA-1273.211 booster dose were also higher than those after a 50-µg mRNA-1273 booster dose (NCT04405076) against the ancestral SARS-CoV-2 and Beta, Omicron BA.1 and Delta variants. The most common local adverse reactions to the bivalent booster injection site are pain, fatigue, headache, and myalgia [23]. Studies have shown the new bivalent boosters to be more effective than the old monovalent boosters in protecting against the Omicron variants. Lin et al. reported that the bivalent booster effectiveness waned at four weeks post vaccination [24]. For all participants aged 12 years and older, vaccine effectiveness against severe infection that required hospitalizations for 15 to 99 days was 25% after receiving a monovalent booster (95% confidence interval (CI), −0.2 to 44.2) [23]. Vaccine effectiveness for all participants that received one bivalent booster dose was 58.7% (95% CI, 43.7 to 69.8). This computes to a difference of 33.5% (95% CI, 2.9 to 62.1) in vaccine effectiveness. Vaccine effectiveness against severe infection resulting in hospitalization or death was 24.9% (95% CI, 1.4 to 42.8) for the monovalent booster and 61.8% (95% CI, 48.2 to 71.8) for one bivalent booster dose, computing to a difference of 36.9% (95% CI, 12.6 to 64.3) [24]. The authors obtained similar results with participants aged 18 years and older or 65 years and older. The Moderna and Pfizer boosters showed similar vaccines effectiveness in all doses examined (first, second, and third booster doses).

## 4. Adverse Events Associated with the Bivalent Omicron Boosters

Reported adverse events associated with the bivalent booster appear consistent with those reported for the monovalent booster; they are less common and less serious than those associated with the COVID-19 illness (Figure 1) [25]. The Vaccine Adverse Events Reporting System (VAERS) report for the period of August 31 to 23 October 2022 included 5542 reports of adverse events after bivalent booster vaccination among persons aged ≥12 years; 95.5% of reports were non serious and 4.5% were serious events [26]. These non-serious events included fatigue (575; 10.9%), pain at the injection site (524; 9.9%), headache (628; 11.9%), fever (561; 10.6%), and chills (459; 8.7%) [26]. Among the 251 that were classified as serious, 4 reports of pericarditis and 5 reports of myocarditis were included. There were 20 reports of individuals who developed COVID-19 disease. There were 36 deaths reported, with a median age of 71 years. Hause et al. reported that during the period of 12 October 2022 to 1 January 2023, 861,251 children aged 5–11 years received the bivalent Pfizer booster, while 92,108 children aged 6–11 years received the bivalent Moderna booster [25]. Among these children, 3259 reported adverse events to VAERS. Both local (68.7%) and systemic reactions (49.5%) were reported. However, 99.8% of the reported cases were classified as being non-serious [26]. Pain at the injection site was the most commonly reported side effect among people vaccinated with the Moderna bivalent omicron booster, according to the CDC. About 80% of trial participants reported it, followed by fatigue, headache, muscle and joint pain, chills, nausea and vomiting, and fever [26].

## 5. Vaccine Hesitancy and the Bivalent Omicron Booster

**Figure 2:**
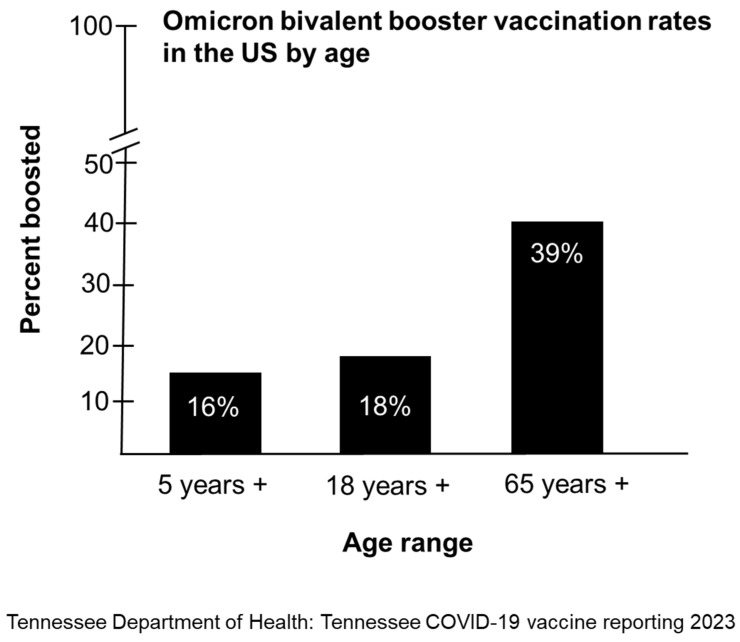
Bivalent booster vaccination rates per 100,000 persons in the US by age group.

The bivalent booster represents the last of the five vaccinations currently recommended for eligible adults and children. Fatigue is likely to be a side effect of concern for those who remain unclear on the need for multiple booster shots after their primary vaccination series. A study by researchers at Duke University, Georgia Tech, and École Normale Supérieure in France explored vaccine hesitancy associated with the updated Omicron booster using data from an opt-in internet survey of 1200 previously vaccinated U.S. adults from November 1 to 5 November 2022. According to survey data, the three most common reasons leading to unwillingness to obtain booster shots were (1) lack of awareness about eligibility for the booster shots (23.2%); (2) lack of awareness about vaccine availability (19.3%); and (3) perceived immunity against infection (18.9%) [27]. Other reasons included (1) concerns regarding the side effects, safety, and effectiveness of the bivalent boosters; (2) insufficient time off from work; and (3) the shots require too much effort to get [27]. In the study, 6 out 10 people stated they did not receive the booster. However, after being informed on booster eligibility and availability, 67.8% of participants who had not yet received the booster said they planned to get it. Finally, for those who had received the booster, the most common reasons reported were (1) they want to protect themselves (90.7%); (2) they want to prevent severe disease (80.6%); and (3) they want to protect others (75%) [27]. Uptake of the bivalent Omicron booster in the US has been poor. In the US, only 16% of children aged 5 years and older, 18% of individuals aged 18 years and older, and 39% of individuals aged 65 years and older have received the updated OBBs (Figure 2). Overall, only 15.8% of individuals in the US have received the updated OBBs.

## 6. Uptake of the Updated OBBs in Tennessee

**Figure 3:**
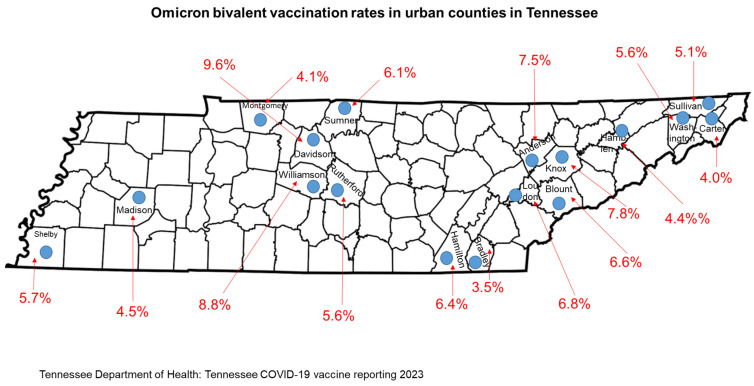
Bivalent booster vaccination rates per 100,000 in Middle Tennessee by age range.

**Figure 4:**
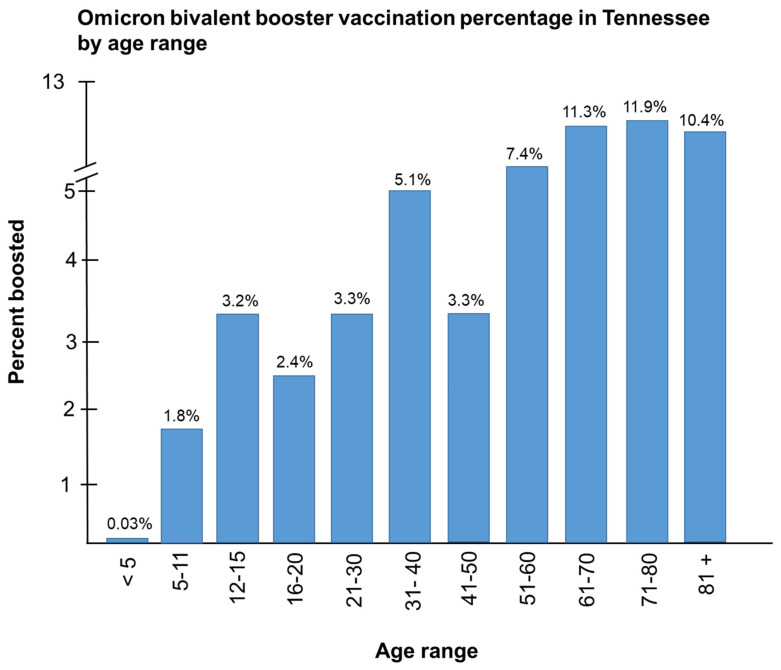
Bivalent booster vaccination rates per 100,000 persons in urban counties in Tennessee. Tennessee Department of Health: Tennessee COVID-19 vaccine reporting https://www.tn.gov/health/cedep/ncov/covid-19-vaccine.html (accessed on 6 February 2023).

**Figure 5:**
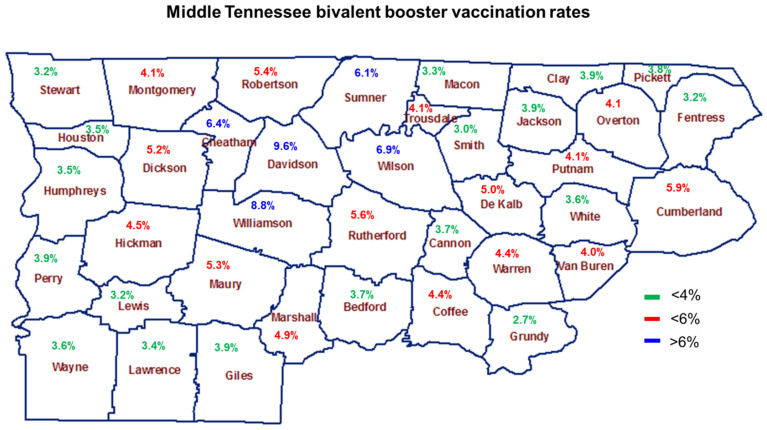
Bivalent booster vaccination rates per 100,000 persons in Middle Tennessee.

The poor uptake of the new OBBs in Tennessee is a microcosm of the South. In Tennessee, we observed the lowest booster vaccination percentages among individuals aged less than 5 years (0.03%) and the highest vaccine uptake among seniors aged 71–80 (11.87%) [15] (Figure 3), both of which are significantly lower than older Tennesseans aged 61–80 years who had the highest vaccination rates per 100,000. We also examined the OBB vaccination rates in the most populous urban counties in Tennessee. With the exception of Bradley County, all urban counties had vaccination rates that were 4% or higher (Figure 4) [5]. Davidson County had the highest vaccination rate at 9.6%, followed by Williamson County at 8.8%, Knox County at 7.8%, and Anderson County at 7.5% [15]. All remaining urban counties registered rates that were below 7.0% (Figure 5). Next, we examined the OBB vaccination rates in Middle Tennessee, which consists of a mixture of urban and rural counties (Figure 5). We observed 18 counties in Middle Tennessee with vaccination rates of less than 4%, 14 counties with rates that were less than 6%, and only 5 counties with rates greater than 6% (Davidson, Williamson, Wilson, Cheatham, and Sumner) (Figure 5) [15]. All county vaccination rates are per 100,000 persons.

### 6.1. Disparities in Uptake of OBBs in Tennessee

**Figure 6:**
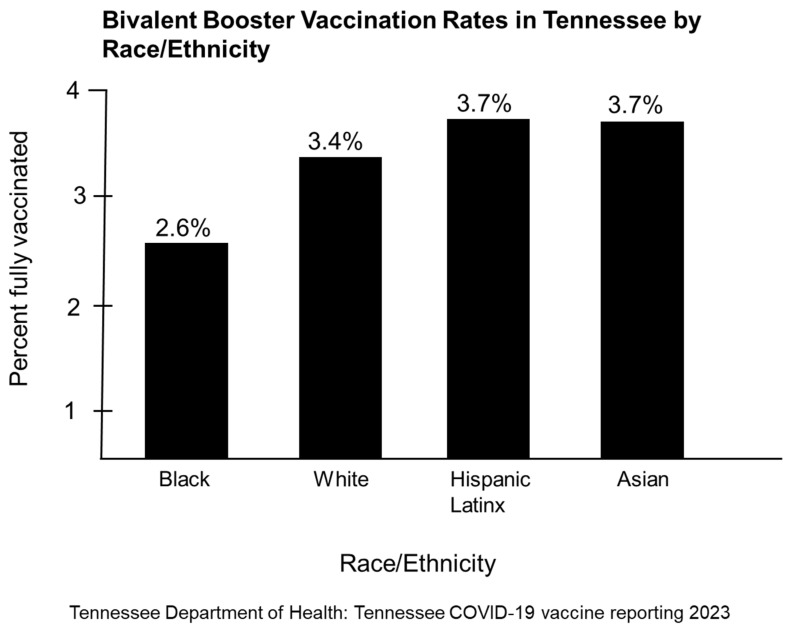
Bivalent booster vaccination rates per 100,000 persons by race/ethnicity. Tennessee Department of Health: Tennessee COVID-19 vaccine reporting 2023.

Looking at OBB vaccination rates in Tennessee by race and ethnicity, we observed the lowest vaccination rate among Blacks (2.6%) followed by Whites (3.4%), Asians (3.4%) and Hispanic/Latinx (3.7%) (Figure 6) [15]. It is unclear if the disparity in the uptake of the OBBs among Blacks is related to education, awareness via public messaging, equity, access, mistrust, misinformation that leads to vaccine hesitancy, health literacy, or vaccine fatigue. Barriers to vaccine access and equity in rural counties can differ from those in urban counties in Tennessee. The bivalent mRNA boosters are widely available in Tennessee except in some rural communities. Some community members may not be aware of the online resources that will provide them with locations for getting the bivalent booster and information about making vaccination appointments at no cost. Moreover, some community members may be unable or unwilling to take time from work and travel long distances to get the new boosters.

### 6.2. The Need for OBBs among Vulnerable Populations

Among individuals in the US age 65 years and older that are vulnerable to the most severe complications of COVID-19, the primary vaccination rate was 94% but the OBB vaccination rate was only 39% [27]. Vulnerable individuals aged 65 years and older often have underlying medical conditions and experience immune senescence due to aging [28]. Therefore, older individuals are likely less able to mount a robust response to COVID-19 vaccines and require up-to-date boosters for the best protection against severe illness, hospitalization, and death. On 11 May 2023, the National COVID-19 Emergency Declarations supported by the Biden administration will end [29]. The Care Plan provided access to COVID-19 testing, treatments, and vaccines at no cost, regardless of health coverage status [29]. The burden of these health care costs will devastate the vulnerable populations and the medically underserved. Communities in need will be forced to forgo COVID-related services and in turn put them at greater risk for severe disease, hospitalizations, and death. All vaccination rates are per 100,000 persons.

### 6.3. Strategies to Improve Vaccine Confidence and Uptake of the OBBs

Evidence-based strategies to improve vaccine uptake should include social, behavioral, communication as well as implementation science approaches to support clinics, individuals and organizations to reach participants experiencing vaccine hesitancy [30]. Evidence-based policy and community-level intervention to improve OBB can include programs that reduce cost for individuals including students, women and children, and reduce barriers to vaccine access in schools and child-care centers [31]. Patient/physician engagement interventions have produced high-level vaccination rates, and for COVID vaccines, primary care physicians are among most trusted messengers for vaccine information [32]. Primary care physicians’ recommendations for vaccines are considered highly reputable and have consistently improved vaccination rates [33,34]. Surveys conducted by Reiter et al., showed that COVID-19 vaccine uptake was more likely when recommended by a clinician [35]. In addition, physicians that employ strong presumptive, announcement-style language rather than conversational, participatory-style language when discussing vaccines can result in improved vaccine uptake [36]. Patient education and awareness strategies to improve vaccine uptake are essential, and patients should understand the benefits gained by vaccinating as opposed to the consequences of failing to vaccinate [37]. In addition, attempts to counter misinformation about OBB should be carefully managed to offer new fact-based information about the vaccine/disease, which can be a more effective approach [38]. Interventions that employ implementation science strategies at the organizational level can include reducing barriers to vaccine and information access especially for marginalized communities, deployment of reminder recall systems, orders for nurse visits, home visits, and point-of-care-prompts [39,40].

Improving vaccine access and equity for underserved communities is essential and may require the deployment of mobile vaccination units and vaccine strike teams that can go door-to-door to provide booster vaccines to at-risk, marginalized populations with historically poor access to medical services and primary care providers in Tennessee.

## 7. Conclusions

The rationale for developing OBBs was strategic, and was based on the continual emergence of the virus and on gauging the ability of vaccine manufacturers to respond to new and future viral emergence using innovative technologies. With waning immunity in the general population vaccinated with ancestral strain vaccines and the emergence of Omicron subvariants, it became clear that an updated vaccine was needed to support public health, especially among vulnerable populations. The OBBs have proven to be safe and more effective than previous boosters and this information should support higher levels of vaccine uptake among vaccine-hesitant populations. All vaccines have side effects and some of these side effects can be severe and immediate such as anaphylaxis; however, most side effects with the OBBs have been found to be mild and similar to the side effects observed with previous Pfizer and Moderna mRNA COVID-19 vaccines. Again, this information should support vaccine confidence and uptake among individuals that are vaccine-hesitant. We also examined strategies to improve vaccine confidence and uptake that include medically underserved and vulnerable populations.

There are high levels of COVID-19 hesitancy in the Southern US. Uptake of the OBBs in Tennessee is at 5.89% for eligible individuals aged five years and older and is below the US national average of 15% [41]. Urban counties in Tennessee have significantly larger populations, higher levels of healthcare infrastructure, more health care providers and liberal political views. Rural counties have smaller populations, lower levels of healthcare infrastructure, less access to healthcare providers and conservative political views. The state data provide aggregate information on vaccine uptake, which is low overall, and looking at county-level data, higher levels of vaccine uptake can be seen in urban counties vs. rural counties, something which has been a trend throughout the pandemic for all COVID-19 vaccines administered.

Although the FDA recently authorized Pfizer’s OBB as the fourth shot for children under 5 years old, OBB vaccination rates among children in this age group have been low (0.03%) per 100,000 persons. The reasons for poor vaccine uptake among this population is unclear. However, the unwillingness of parents to provide consent for this age group out of fear and misinformation greatly contributes. In addition, EUA eligibility requires that children 6 months through 4 years of age who completed their three-dose primary series with the monovalent Pfizer COVID-19 vaccine more than two months previously are now eligible to receive the Pfizer OBB. Many children in this age have not received any vaccines and may not meet eligibility requirements. As of 14 March 2023, the OBB is authorized in the European Union (EU) as a booster dose for ages 5 years and older. The OBB is approved via EUA by the FDA and is not approved currently for use in other countries. Full approval of the OBB in the future is unclear. There is currently no data in the literature on use of the OBB outside of the US at this time.

At the time of writing (14 February 2023), Tennessee is experiencing a surge in COVID-19 cases and hospitalizations [28]. The OBBs from Pfizer and Moderna have been shown clinically to be safe and more effective than the previous monovalent boosters. Adverse events associated with the OBB boosters reported to the VARES have been overwhelmingly classified as non-serious, but rare, serious, and life-threatening adverse events do occur. Data from the Tennessee Department of Health show a disparity in the uptake of OBBs among Blacks compared to other major ethnic groups. The reasons for this disparity are unclear and are likely multifactorial. We are particularly concerned about vulnerable minority populations that have traditionally had poor access to COVID-19 vaccines. With the ending of the National COVID-19 Emergency Declarations in the US on 11 May 2023, medically underserved communities without health insurance will have to pay out-of-pocket for COVID-19 testing, treatments, and vaccines. These services will likely be cost prohibitive for these communities, further increasing the COVID-19 disease burden, hospitalizations, and deaths among these communities.

Limitations of this review include the focus on the state of Tennessee due to the poor uptake of the OBB during a time when COVID-19 was surging in Tennessee alongside Tennessee’s ranking in COVID-19 cases and deaths compared to other states in the US. In addition, the concentration on Middle Tennessee due to its population, size, and unique mixture of both urban and rural counties could be seen as a limitation. The review was not designed to be a population-based study on OBB vaccine uptake on a global scale because of limited availability outside of the US.

Taken together, all eligible individuals including children under 5 years to 6 months old should receive a bivalent booster vaccine as recommended and health professionals, including primary care providers, should employ evidence-based strategies to inform the public about the importance of this newly developed and safe vaccine. Understanding OBB vaccine barriers in a climate of vaccine fatigue is critical because an mRNA COVID-19 booster will likely be included as an annually scheduled vaccination in the US. The initial poor uptake of the OBB vaccine in Tennessee and throughout the US suggests that the immune protection barrier that has been established could be lost over time because of waning immunity and immune senescence in the elderly, resulting in a large at-risk-population that would be highly vulnerable to newly emerged subvariants. Surveillance of SARS-CoV2 emergence over time may require timely development of novel mRNA vaccines or even future combinatorial vaccines to ensure public health safety. Development of the OBB vaccine supports the notion that manufacturers can respond to the emergent challenges of the COVID-19 pandemic to support public health. The pandemic is in a different phase at this time due to large scaled vaccinations and the recent emergence of variants that are highly contagious but less pathogenic than previous variants. However, hospitalizations and deaths still occur mainly among the elderly and individuals with underlying medical conditions. Timely uptake of the OBB vaccine may require a national campaign in partnership with the federal government and the CDC. Now that SARS-CoV-2 is endemic, it is critical that we continue to develop safe and effective COVID-19 vaccines.

## Data Availability

This manuscript did not report any laboratory-based data.
